# A novel immune-related gene signature predicts the prognosis of hepatocellular carcinoma

**DOI:** 10.3389/fonc.2022.955192

**Published:** 2022-09-15

**Authors:** Shujiao He, Jingqiao Qiao, Lei Wang, Li Yu

**Affiliations:** Department of Hematology and Oncology, International Cancer Center, Shenzhen University General Hospital, Shenzhen University Health Science Center, Shenzhen, China

**Keywords:** hepatocellular carcinoma, expression landscape, differentially expressed genes, immune-related genes, prognostic signature

## Abstract

Immune-related genes play a key role in regulating the cancer immune microenvironment, influencing the overall survival of patients with hepatocellular carcinoma (HCC). Along with the rapid development of immunotherapy, identifying immune-related genes with prognostic value in HCC has attracted increasing attention. Here, we aimed to develop a prognostic signature based on immune-related genes. By investigating the transcriptome landscape of 374 HCC and 160 non-HCC samples *in silico*, a total of 2251 differentially expressed genes were identified. Among which, 183 differentially expressed immune-related genes were subjected to a univariate Cox proportional hazard model to screen for genes with possible prognostic significance. A 10-gene prognostic signature, including *HLA-G*, *S100A9*, *S100A10*, *DCK*, *CCL14*, *NRAS*, *EPO*, *IL1RN*, *GHR* and *RHOA*, was generated employing a multivariate Cox proportional hazard model. Kaplan–Meier and Receiver Operator Characteristic (ROC) curves were used to evaluate the prognostic utility of the 10-gene signature. Moreover, the underlying mechanisms of these genes were analyzed *via* Gene Ontology (GO) and Kyoto Encyclopedia of Genes and Genomes (KEGG) enrichment. According to the Tumor Immune Estimation Resource (TIMER) database, our prognostic signature was significantly associated with tumor-infiltrating B cells, CD4 T cells, dendritic cells, macrophages and neutrophils. Our study provides a novel prognostic signature based on immune-related genes associated with clinical outco mes of HCC.

## Introduction

Hepatocellular carcinoma (HCC) is a highly heterogeneous and lethal malignancy which ranks as the second leading cause of cancer-related death worldwide (1, 2). Despite considerable efforts to improve clinical outcomes, the prognosis remains unsatisfactory. The Barcelona Clinic Liver Cancer (BCLC) remains the most widely used evaluation system for predicting HCC in clinical practice (3). It focuses primarily on a subset of patients who may benefit from ablative or intra-arterial treatment, which may not meet the predictable need for innovative treatment (4). Therefore, there is still a need for an optimized prediction system to guide clinical treatment.

Over the past decade, immunotherapy has become one of the most promising cancer treatments (5). Immune checkpoint inhibitors, which are essential components of immunotherapy, play a significant role in the treatment of advanced HCC (6). Programmed cell death protein 1 (PD-1) monoclonal antibodies, such as nivolumab and pembrolizumab, have already been approved by the Food and Drug Administration (FDA) for the treatment of advanced HCC (7). Nivolumab and pembrolizumab treatment resulted in a 2-year survival rate of 80% for patients with advanced HCC. Additionally, cytotoxic T-lymphocyte-associated protein 4 (CTLA-4) blockade with tremelimumab induced sustained objective remission in patients with HCC who were also diagnosed with hepatitis C virus (HCV) (8). However, poor clinical responses to immunotherapy are still common due to limited infiltration of immune checkpoint inhibitors and the impaired anti-tumor effect of CD8+T cells in the tumor microenvironment (9). Immune-related genes that participate in immunotherapy resistance and tumor progression (10) play a central role in modulating the tumor immune microenvironment (11, 12). Therefore, developing a prognostic monitoring system based on immune-related genes would provide a precise prediction model for patients and insight into new therapeutic approaches.

## Materials and methods

### Dataset acquisition and pre-processing

The Level-3 gene expression data and the corresponding clinical characteristics of HCC patients were obtained from The Cancer Genome Atlas (TCGA) portal (https://portal.gdc.cancer.gov/). Additionally, a normal liver transcriptome dataset derived from the Genotype-Tissue Expression Project (GTEx) was obtained from the University of California, Santa Cruz (UCSC) Xena database (http://xena.ucsc.edu/). All raw data were annotated according to the human genome annotation file (GRCh38.p12) obtained from Ensembl (http://asia.ensembl.org/Homo_sapiens/Info/Index). Following normalization using the same criteria, log2(FPKM+1), the raw datasets from TCGA and GTEx were merged to form a study project with 374 HCC samples and 160 non-tumor samples. A subset of immune-related genes was downloaded from the ImmPort database (https://www.immport.org/shared/home). The characteristics of tumor-infiltrating immune cells were retrieved from the Tumor Immune Estimation Resource (TIMER, https://cistrome.shinyapps.io/timer/) database, which was compiled based on the TCGA data.

### Differentially expressed gene analysis

Differentially expressed genes (DEGs) between HCC tumor and non-tumor samples were obtained using the EdgeR package (13) in R software (version 3.6.1) (14). Genes were considered DEGs if they met the following screening criteria: false discovery rate (FDR) < 0.05 and a log_2_ |fold change| > 1. Differentially expressed immune-related genes (DEIGs) were extracted from DEGs by intersecting them with the immune-related gene dataset.

### Development of a prognostic model and analysis

The primary endpoint of the study was death. Patients with a survival time of less than 30 days or those lost to follow-up were censored (15). A total of 342 patients with HCC were subjected to univariate Cox analysis to identify genes associated with survival. The identified DEIGs were considered hub genes. Statistical analyses were conducted *via* the survival package (16) in R. A value of *p <* 0.01 was considered statistically significant.

Hub genes were subsequently subjected to a multivariate Cox regression analysis to construct a prognostic model. A receiver operator characteristic (ROC) curve was developed to assess the performance of the prognostic model. The prognostic model and clinical parameters (age, gender, the grade and stage of disease) were subjected to univariate and multivariate analyses to identify the independent prognostic factors. Genes that were eligible to construct the prognostic model were identified as key genes.

### Survival analysis

All patients with HCC were allocated with a risk score according to the prognostic risk model formula, then the median value of the risk scores was employed as the cutoff. Patients with a risk score higher than the median value were considered the high-risk group and patients with a risk score lower than the median value were considered the low-risk group. We then compared the mortality rates of the two groups by R survival (16) and limma packages (17). Univariate survival analysis was performed for each of the key genes within the prognostic model. The median expression level was used to divide the patients into high- and low-expression groups.

### Gene enrichment analysis

The Gene Ontology (GO) and Kyoto Encyclopedia of Genes and Genomes (KEGG) functional enrichment analyses were conducted to explore the possible molecular mechanisms of the DEIGs and the biological functions of the 10 key genes in the signature.

### Correlation analysis

The relationship between the prognostic model and the infiltration level of immune cells in HCC samples was analyzed and visualized *via* R. The *p-value* was set to *p <* 0.05 as significance level and the absolute value of the correlation coefficient was set to < 0.3 as the cutoff values.

## Results

### Identification of DEIGs

**Figure 1:**
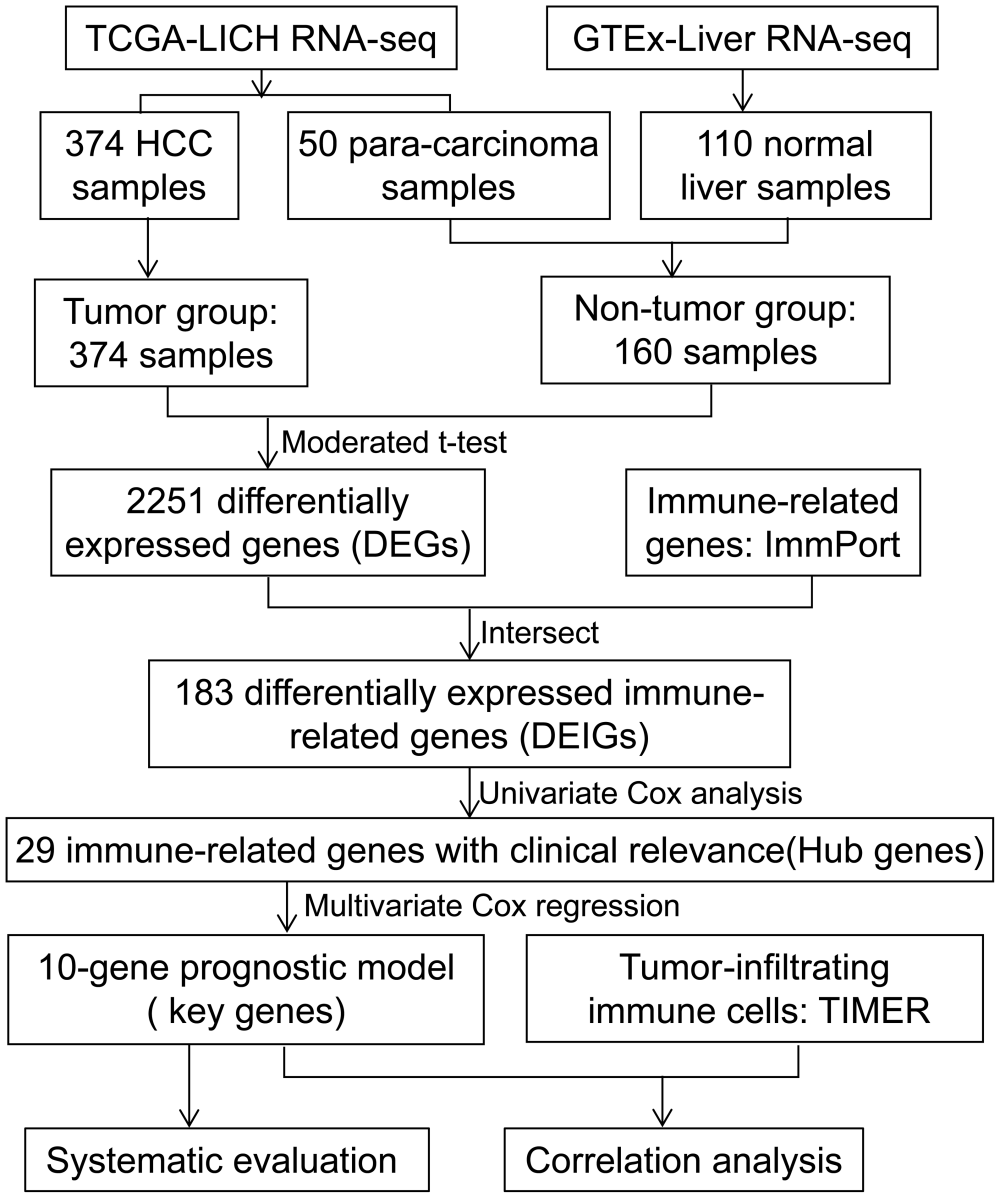
Flow diagram of the study.

**Figure 2:**
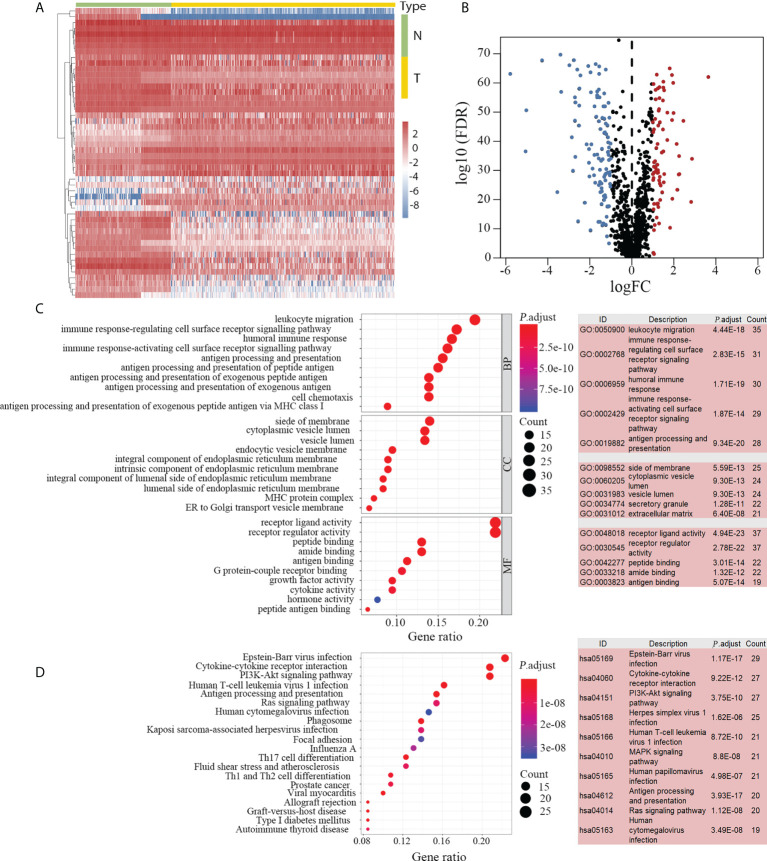
DEIGs between 374 HCC and 160 non-tumor samples. DEIGs between tumor and non-tumor groups are depicted in the heatmap **(A)** and volcano plot **(B)**. Among the 183 identified DEIGs, 75 genes were upregulated and 108 genes were downregulated in HCC. GO enrichment analysis revealed 180 enriched DEIGs (**C**, left). The top five GO items with the most enriched genes are listed (**C**, right). KEGG enrichment analysis revealed 130 enriched DEIGs (**D**, left) and the 10 KEGG items with the most enriched genes are listed (**D**, right). DEIGs, differentially expressed immune-related genes; HCC, hepatocellular carcinoma; GO, gene ontology; KEGG, Kyoto Encyclopedia of Genes and Genomes; N, non-tumor group; T, tumor group; BP, biological processes; CC, cellular components; MF, molecular functions.

A flow diagram of the present study is presented in **Figure 1**. The tumor group consisted of 374 HCC samples obtained from TCGA, and the non-tumor group included 50 paracarcinoma samples from TCGA and 110 normal liver samples from GTEx. We identified 2251 DEGs by comparing the expression profiles of the tumor and non-tumor groups. There were 880 upregulated and 1371 downregulated DEGs observed in HCC. By intersecting immune-related genes with DEGs, 183 DEIGs were identified, of which 75 were upregulated in HCC (**Figures 2A, B**).

Subsequent enrichment analyses indicated that 180 and 130 DEIGs were enriched by GO (**Figure 2C**) and KEGG (**Figure 2D**) analyses, respectively. The inflammatory related items were most frequently mentioned in the enrichment analyses. In the GO enrichment analysis, “leukocyte migration”, “side of membrane” and “receptor-ligand activity” were the most frequent biological terms among “biological processes (BP)”, “cellular components (CC)” and “molecular functions (MF)” categories, respectively (**Figure 2C**). In the KEGG enrichment analysis, “Epstein-Barr virus infection” was the most predominant item that significantly enriched 29 DEIGs (**Figure 2D**).

### Construction of the prognostic model

**Figure 3:**
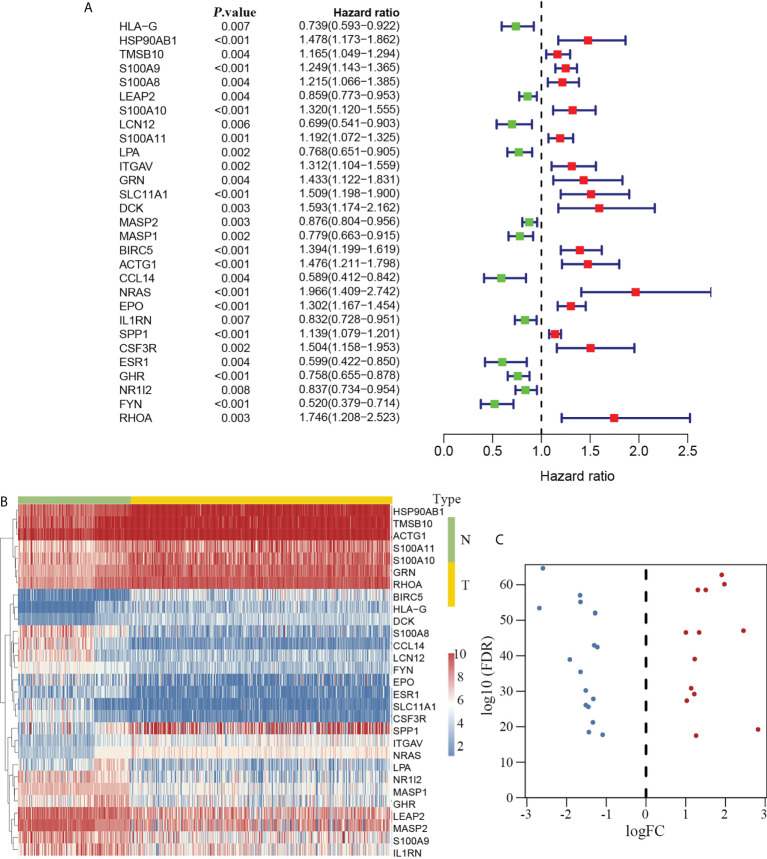
Immune-related hub genes in HCC. Twenty-nine DEIGs were identified that were associated with clinical outcomes in HCC (**A**, left). Twelve had an HR less than 1.0 and were considered protective factors, whereas 17 genes had an HR greater than 1.0 (**A**, right) and were regarded as risk factors. The heatmap delineating the expression profiles of the 29 hub genes **(B)**. The volcano plot showing that 13 hub genes were upregulated and 16 genes were downregulated in HCC **(C)**. DEIGs, differentially expressed immune-related genes; HCC, hepatocellular carcinoma; HR, hazard ratio; N, non-tumor group; T, tumor group.

Twenty-nine immune-related genes were identified as hub genes with clinical significance in HCC according to the univariate Cox proportional hazard model. Among these genes, 12 genes with a hazard ratio (HR) less than 1.0 were regarded as protective factors, whereas 17 genes with an HR greater than 1.0 were considered as risk factors (**Figure 3A**). We compared the expression disparity of the 29 hub genes between tumor and non-tumor tissues. The results showed that 13 hub genes were upregulated and 16 were downregulated in HCC (**Figures 3B, C**).

Subsequently, the hub genes were subjected to multivariate Cox analysis to construct a prognostic risk score model. Ten hub genes were identified as key genes for developing the prognostic model. According to the model formula, each patient was labelled with a risk score. The risk model is as follows:

Risk score = [Expression level of HLA-G* (-0.38009)] + [Expression level of S100A9* 0.23467] + [Expression level of S100A10* 0.14180] + [Expression level of DCK* 0.81204] + [Expression level of CCL14* 0.52696] + [Expression level of NRAS* 0.59515] + [Expression level of EPO*0.24953] + [Expression level of IL1RN* (-0.18841)] + [Expression level of GHR* (-0.30230)] + [Expression level of RHOA* (-0.43270)].

### Verification of the prognostic model

**Figure 4:**
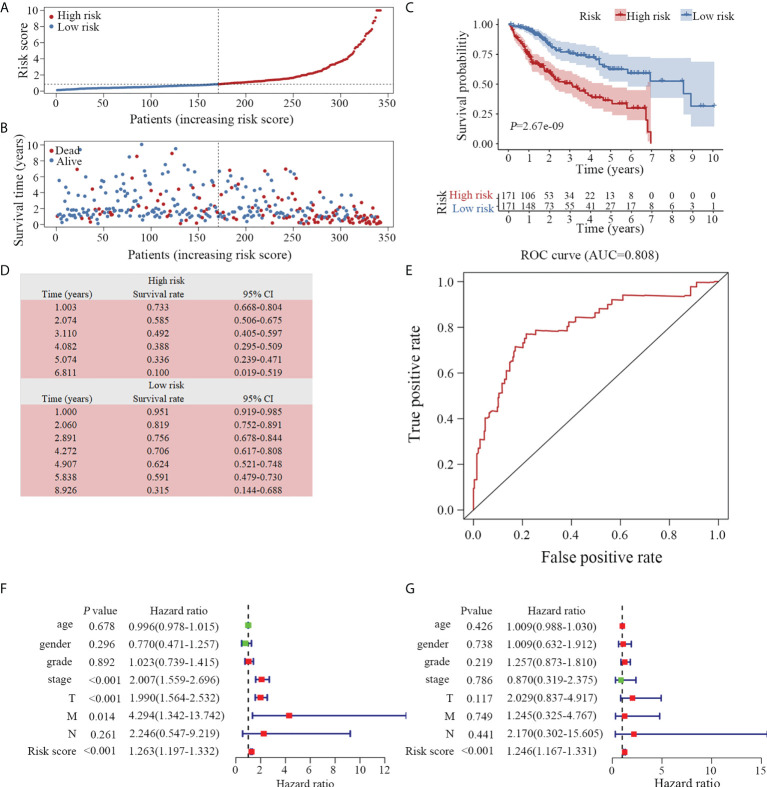
Evaluation of the prognostic model. **(A)** Distribution of 342 patients with HCC according to the prognostic model. **(B)** Survival status based on the risk score of the prognostic model. Patients with a low-risk score exhibited a longer survival time. **(C)** Based on the survival curves, the OS was significantly lower in the high-risk than in the low-risk groups. **(D)** The survival rate of high- and low-risk groups is listed. **(E)** According to the ROC curve, the clinical utility of the model is validated (AUC=0.808). Both univariate **(F)** and multivariate **(G)** Cox analyses revealed that the prognostic model was an independent predictive indicator for HCC. OS, overall survival; ROC, receiver operator characteristic; AUC, area under curve. TNM, Tumor-Node-Metastasis staging.

To investigate the prognostic value of the model, we divided the 342 enrolled patients with HCC into high- and low-risk groups based on the median value of the risk scores according to the prognostic risk model (**Figure 4A**). A higher risk score indicated a shorter survival time (**Figure 4B**). Additionally, patients in the low-risk group had significantly longer survival times than those in the high-risk group (**Figure 4C**). The five-year survival rate of the high- and low-risk groups were 33.6% and 62.4%, respectively (**Figure 4D**). In addition, we assessed the predictive ability of the prognostic model using the ROC curve. The area under the curve (AUC) was 0.808 (**Figure 4E**), indicating that the prediction model was highly accurate.

To determine whether the model is an independent predictor of prognosis, we examined the prognostic model along with commonly used clinical parameters, including age, sex, grade and cancer stage of the patients. A prognostic analysis was conducted on 221 patients with complete clinical data *via* Cox regression analysis. In conjunction with both univariate (**Figure 4F**) and multivariate (**Figure 4G**) Cox analyses, the prognostic model was demonstrated to be a reliable and independent indicator of HCC clinical outcomes.

### Estimation of tumor infiltrating immune cells in HCC samples

**Figure 5:**
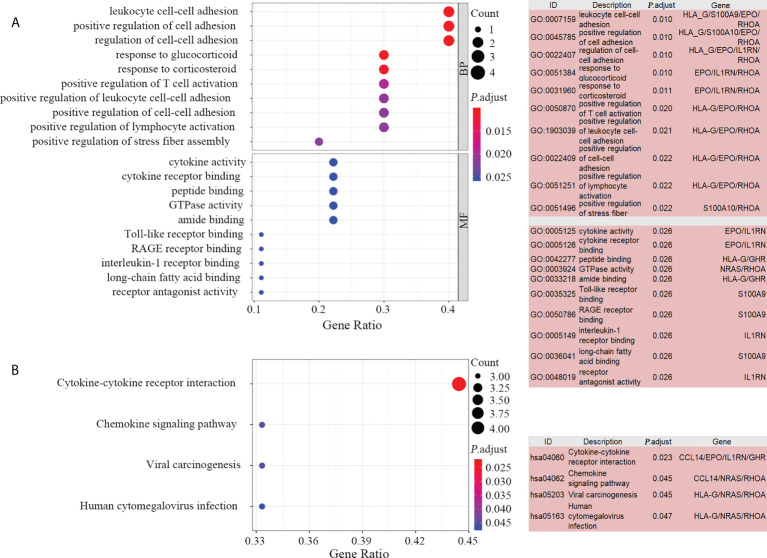
Enrichment analysis of the 10 key genes. Bubble plot (**A**, left) and list box (**A**, right) show the top 10 GO items for BP and MF. Significant KEGG pathways of the 10 key genes visualized by bubble plot (**B**, left) and list box (**B**, right). GO, gene ontology; KEGG, Kyoto Encyclopedia of Genes and Genomes; BP, biological processes, MF, molecular functions.

The mechanisms and pathways involved in the prognostic model were depicted by performing 10 key gene-based enrichment analyses. GO enrichment analysis indicated that no genes were enriched in the GO-CC item. The most frequently occurring items in GO-BP and GO-MF were “leukocyte cell-cell adhesion” and “cytokine activity”, respectively (**Figure 5A**). In the GO-term enrichment analysis, *RHOA*, *EPO* and *HLA-G* were the most widespread genes, found in 119, 69 and 59 terms, respectively. In KEGG analysis, the pathway “cytokine-cytokine receptor interaction” was the most significant pathway (**Figure 5B**) and *RHOA* and *NRAS* were the most abundant key genes.

**Figure 6:**
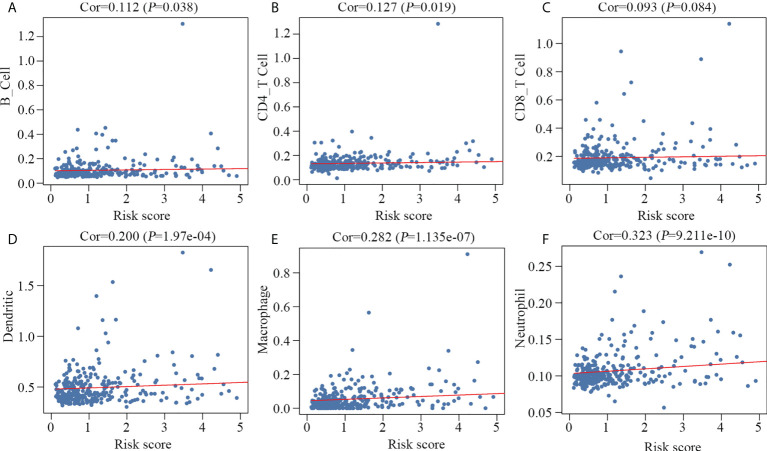
Relationship between the prognostic model and the infiltration level of six immune cell types. B cells **(A)**, CD4 T cells **(B)**, dendritic cells **(D)**, macrophages **(E)** and neutrophils **(F)** were significantly correlated with the prognostic model, but not CD8 T cells **(C)**.

We speculated that the 10 key genes contribute to modulating the tumor immune microenvironment. Therefore, we examined the correlation between the prognostic model and the infiltration level of the six immune cells. Our results suggested a positive correlation between the prognostic model and B cells, CD4 T cells, dendritic cells, macrophages and neutrophils (**Figures 6A, B, D–F**) in HCC. There was no correlation between the prognostic model and the presence of tumor infiltrating CD8 T cells (**Figure 6C**).

### Signatures of the 10 key genes

**Figure 7:**
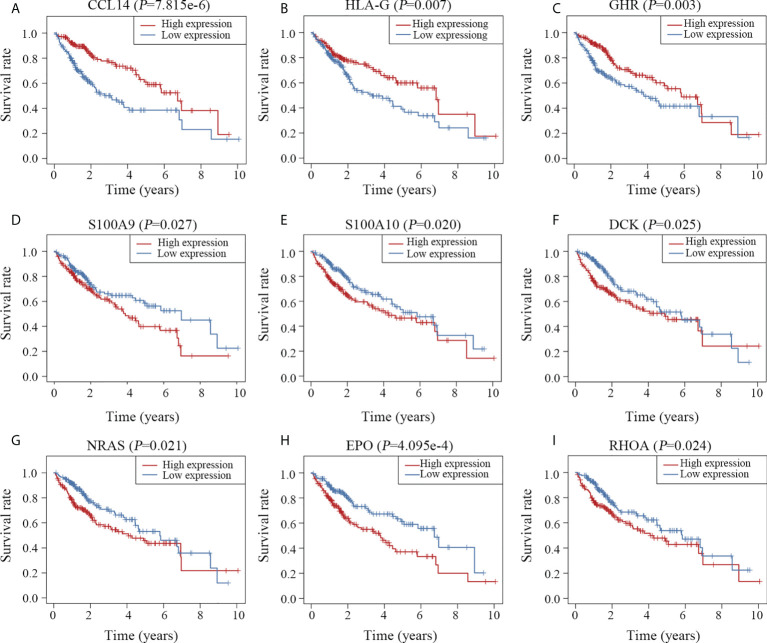
Survival curves of the 10 key genes. Patients with high expression of *CCL14* **(A)**, *HLA-G* **(B)** and *GHR* **(C)** survived longer than those with low expression of these genes. High expression of *S100A9* **(D)**, *S100A10* **(E)**, *DCK* **(F)**, *NRAS* **(G)**, *EPO* **(H)** and *RHOA* **(I)** was associated with poor clinical outcomes.

Univariable survival analysis was carried out for all genes involved in the prognostic model to gain further insight into the components of the predictive model. Patients were divided into high- and low-expression groups based on the median values of normalized expression levels. Except for *IL1RN*, nine other genes were significantly associated with the overall survival time of patients with HCC (**Figure 7**). In accordance with the results of the Cox analysis (**Figure 3A**), patients with high expression levels of *CCL14*, *HLA-G* and *GHR* had longer survival times (**Figures 7A–C**), whereas those with high expression levels of *S100A9*, *S100A10*, *DCK*, *NRAS*, *EPO* and *RHOA* had shorter survival times (**Figures 7D–I**).

## Discussion

HCC is a highly heterogeneous disease with poor clinical outcomes. With the advent of immunotherapy in malignant diseases, immune-related genes have been of interest to the research community in recent years. In this study, we constructed an immune-related 10-gene model to predict HCC prognosis by mining public datasets from TCGA and GTEx.

We collected transcriptome data from 374 HCC and 50 para-tumor samples from TCGA. To reduce the bias in the analysis caused by the disparity in sample sizes, expression profiles of 110 normal liver samples from GTEx were added to the non-tumor group. As HCC often occurs in the context of cirrhosis and hepatitis (18), the transcriptome profiles of para-tumors may differ significantly from those of normal liver tissues. Adding normal liver samples to the non-tumor group provides a better analysis strategy with improved validity and reliability.

Following the acquisition of DEIGs by intersecting DEGs with immune-related genes, univariate Cox analysis was conducted to identify immune-related genes that were associated with clinical prognosis. A 10-gene prognostic model was developed and evaluated using univariate and multivariate Cox proportional hazard models, Kaplan–Meier estimates and ROC curves. According to the evaluation, the 10-gene model was capable of reliably predicting HCC outcomes.

The prognostic model was constructed based on expression signature of the10 genes, including *CCL14*, *HLA-G*, *GHR*, *S100A9*, *S100A10*, *DCK*, *NRAS*, *EPO*, *RHOA* and *IL1RN*, which was quite different from the previous studies that focused on DEGs (19, 20). Patients with HCC with high *CCL14*, *HLA-G* and *GHR* expression had significantly better outcomes. This is in accordance with other studies demonstrating that *CCL14* (1, 21, 22), *HLA-G* (23, 24) and *GHR* (25) are potential tumor suppressors in HCC. High expression levels of *S100A9* (26, 27), *S100A10* (28), *DCK* (29), *NRAS* (30, 31), *EPO* (32, 33) and *RHOA* (34, 35) were associated with poor prognosis. The relationship between *IL1RN* and clinical outcomes is unclear and little is known about its biological function in HCC.

Furthermore, we validated the close correlation between the prognostic model and the level of immune-infiltrating cells. According to the TIMER database (36), B cells, CD4 T cells, dendritic cells, macrophages, and neutrophils had significant positive correlations with the prognostic model. Although the immune context of HCC is far more complex, the relationship between the prognostic model and the above five types of immune cells provides insight into the regulation of the tumor immune microenvironment.

It is noteworthy that the 10 key genes identified in this study were predominantly involved in ligand-receptor mechanisms for regulating the tumor immune microenvironment. Specifically, CCL14 (C-C motif chemokine ligand 14) is a ligand of CCR1 and CCR5 (37). HLA-G is a ligand of ILT2, ILT4 and KIR2DL4 (38). GHR is a well-known receptor for growth hormones (39). EPO is a ligand of EPOR. IL1RN is a natural interleukin 1 receptor antagonist that inhibits IL-1 activity (40). The ligand-receptor pattern appears to be a promising target for cancer immunotherapy. In summary, the prognostic effect of the immune-related predictors offers novel insights into the tumor immune microenvironment, however, a comprehensive study is still required.

This study has certain limitations that should be addressed. The prognostic risk model must be validated in an independent clinical cohort. Furthermore, the sensitivity and specificity of our prognostic immune biomarkers should be examined both *in vitro* and *in vivo*. Finally, this study concentrated on transcriptome expression profiles, therefore, it cannot reflect the full landscape of immune-related genes in HCC.

In conclusion, using transcriptome profiles of immune-related genes, we developed a reliable model for predicting survival outcomes in patients with HCC. Additionally, as an independent prognostic factor, the model predicted the abundance of immune-infiltrating cells in HCC.

## Data availability statement

The original contributions presented in the study are included in the article/**Supplementary Material**, further inquiries can be directed to the corresponding author/s.

## Author contributions

L.Y designed, guided the study; SJ.H analyzed data and drafted the manuscript; JQ.Q and L.W acquired the data. All authors contributed to the article and approved the submitted version.

## Funding

National Natural Science Foundation of China (82030076, 82070161, 81970151, 81670162 and 81870134). Shenzhen Science and Technology Foundation (JCYJ20190808163601776 and JCYJ20200109113810154). Shenzhen Key Laboratory Foundation (ZDSYS20200811143757022). Sanming Project of Shenzhen (SZSM202111004).

## Acknowledgments

The data supporting this publication is available at UCSC Xena (http://xena.ucsc.edu/), Ensembl (http://asia.ensembl.org/Homo_sapiens/Info/Index), ImmPort (https://www.immport.org) and TIMER (https://cistrome.shinyapps.io/timer/).

## Conflict of interest

The authors declare that the research was conducted in the absence of any commercial or financial relationships that could be construed as a potential conflict of interest.

## Publisher’s note

All claims expressed in this article are solely those of the authors and do not necessarily represent those of their affiliated organizations, or those of the publisher, the editors and the reviewers. Any product that may be evaluated in this article, or claim that may be made by its manufacturer, is not guaranteed or endorsed by the publisher.

## Abbreviations

HCC, Hepatocellular Carcinoma; ROC, Receiver Operator Characteristic; GO, Gene Ontology; KEGG, Kyoto Encyclopedia of Genes and Genomes; TIMER, Tumor Immune Estimation Resource; BCLC, Barcelona Clinic Liver Cancer; FDA, Food and Drug Administration; DEGs, Differentially expressed genes; DEIGs, Differentially expressed immune-related genes; HR, Hazard ratio; AUC, Area under the curve.

## References

[1] Zhu M Xu W Wei C Huang J Xu J Zhang Y . CCL14 serves as a novel prognostic factor and tumor suppressor of HCC by modulating cell cycle and promoting apoptosis. Cell Death Dis (2019) 10(11):796. doi: 10.1038/s41419-019-1966-6 31641099 PMC6805940

[2] Dinarello CA . Biologic basis for interleukin-1 in disease. Blood (1996) 87(6):2095–147. doi: 10.1182/blood.V87.6.2095.bloodjournal8762095 8630372

[3] Vogel A Cervantes A Chau I Daniele B Llovet J Meyer T . Hepatocellular carcinoma: ESMO clinical practice guidelines for diagnosis, treatment and follow-up. Ann oncology: Off J Eur Soc Med Oncol (2018) 29(Suppl 4):iv238–55. doi: 10.1093/annonc/mdy308 30285213

[4] Cai J Tong Y Huang L Xia L Guo H Wu H . Identification and validation of a potent multi-mRNA signature for the prediction of early relapse in hepatocellular carcinoma. Carcinogenesis (2019) 40(7):840–52. doi: 10.1093/carcin/bgz018 31059567

[5] Shimizu Y Suzuki T Yoshikawa T Endo I Nakatsura T . Next-generation cancer immunotherapy targeting glypican-3. Front Oncol (2019) 9:248. doi: 10.3389/fonc.2019.00248 31024850 PMC6469401

[6] Obeid JM Kunk PR Zaydfudim VM Bullock TN Slingluff CL Jr. Rahma OE . Immunotherapy for hepatocellular carcinoma patients: is it ready for prime time? Cancer Immunology Immunotherapy: CII (2018) 67(2):161–74. doi: 10.1007/s00262-017-2082-z PMC11028155 29052780

[7] Mahipal A Tella SH Kommalapati A Lim A Kim R . Immunotherapy in hepatocellular carcinoma: Is there a light at the end of the tunnel? Cancers (2019) 11(8):1078. doi: 10.3390/cancers11081078 PMC6721326 31366113

[8] Giraud J Chalopin D Blanc JF Saleh M . Hepatocellular carcinoma immune landscape and the potential of immunotherapies. Front Immunol (2021) 12:655697. doi: 10.3389/fimmu.2021.655697 33815418 PMC8012774

[9] Bresin A D'Abundo L Narducci MG Fiorenza MT Croce CM Negrini M . TCL1 transgenic mouse model as a tool for the study of therapeutic targets and microenvironment in human b-cell chronic lymphocytic leukemia. Cell Death Dis (2016) 7:e2071. doi: 10.1038/cddis.2015.419 26821067 PMC4816192

[10] Corapi E Carrizo G Compagno D Laderach D . Endogenous galectin-1 in T lymphocytes regulates anti-prostate cancer immunity. Front Immunol (2018) 9:2190. doi: 10.3389/fimmu.2018.02190 30319642 PMC6169479

[11] Schulz M Salamero-Boix A Niesel K Alekseeva T Sevenich L . Microenvironmental regulation of tumor progression and therapeutic response in brain metastasis. Front Immunol (2019) 10:1713. doi: 10.3389/fimmu.2019.01713 31396225 PMC6667643

[12] Zhang X Li X Xie J Zhu Q Yuan Y . A novel immune-related prognostic signature predicting survival in patients with pancreatic adenocarcinoma. J Oncol (2022) 2022:8909631. doi: 10.1155/2022/8909631 35342420 PMC8956421

[13] Robinson MD McCarthy DJ Smyth GK . edgeR: a bioconductor package for differential expression analysis of digital gene expression data. Bioinf (Oxford England) (2010) 26(1):139–40. doi: 10.1093/bioinformatics/btp616 PMC2796818 19910308

[14] R Core Team . R: A language and environment for statistical computing. Vienna, Austria: R Foundation for Statistical Computing (2019). Available at: https://www.R-project.org/.

[15] Wang Q Xia D Bai W Wang E Sun J Huang M . Development of a prognostic score for recommended TACE candidates with hepatocellular carcinoma: A multicentre observational study. J Hepatol (2019) 70(5):893–903. doi: 10.1016/j.jhep.2019.01.013 30660709

[16] Therneau T . A package for survival analysis in r. r package version 3.3-1 (2022). Available at: https://CRAN.R-project.org/package=survival.

[17] Ritchie ME Phipson B Wu D Hu Y Law CW Shi W . Limma powers differential expression analyses for RNA-sequencing and microarray studies. Nucleic Acids Res (2015) 43(7):e47. doi: 10.1093/nar/gkv007 25605792 PMC4402510

[18] Unfried JP Serrano G Suarez B Sangro P Ferretti V Prior C . Identification of coding and long noncoding RNAs differentially expressed in tumors and preferentially expressed in healthy tissues. Cancer Res (2019) 79(20):5167–80. doi: 10.1158/0008-5472.CAN-19-0400 31387921

[19] Long J Zhang L Wan X Lin J Bai Y Xu W . A four-gene-based prognostic model predicts overall survival in patients with hepatocellular carcinoma. J Cell Mol Med (2018) 22(12):5928–38. doi: 10.1111/jcmm.13863 PMC6237588 30247807

[20] Zhou T Cai Z Ma N Xie W Gao C Huang M . A novel ten-gene signature predicting prognosis in hepatocellular carcinoma. Front Cell Dev Biol (2020) 8:629. doi: 10.3389/fcell.2020.00629 32760725 PMC7372135

[21] Zhang X Wan JX Ke ZP Wang F Chai HX Liu JQ . TMEM88, CCL14 and CLEC3B as prognostic biomarkers for prognosis and palindromia of human hepatocellular carcinoma. Tumor biology: J Int Soc Oncodevelopmental Biol Med (2017) 39(7):1010428317708900. doi: 10.1177/1010428317708900 28718365

[22] Gu Y Li X Bi Y Zheng Y Wang J Li X . CCL14 is a prognostic biomarker and correlates with immune infiltrates in hepatocellular carcinoma. Aging (2020) 12(1):784–807. doi: 10.18632/aging.102656 31927532 PMC6977663

[23] Bian X Si Y Zhang M Wei R Yang X Ren H . Down-expression of miR-152 lead to impaired anti-tumor effect of NK *via* upregulation of HLA-G. Tumor biology: J Int Soc Oncodevelopmental Biol Med (2016) 37(3):3749–56. doi: 10.1007/s13277-015-3669-7 26468017

[24] Lin A Chen HX Zhu CC Zhang X Xu HH Zhang JG . Aberrant human leucocyte antigen-G expression and its clinical relevance in hepatocellular carcinoma. J Cell Mol Med (2010) 14(8):2162–71. doi: 10.1111/j.1582-4934.2009.00917.x PMC3823007 19799650

[25] Qi HL Li CS Qian CW Xiao YS Yuan YF Liu QY . The long noncoding RNA, EGFR-AS1, a target of GHR, increases the expression of EGFR in hepatocellular carcinoma. Tumor Biol J Int Soc Oncodevelopmental Biol Med (2016) 37(1):1079–89. doi: 10.1007/s13277-015-3887-z 26271667

[26] Meng J Gu F Fang H Qu B . Elevated serum S100A9 indicated poor prognosis in hepatocellular carcinoma after curative resection. J Cancer (2019) 10(2):408–15. doi: 10.7150/jca.28409 PMC6360318 30719134

[27] Duan L Wu R Zhang X Wang D You Y Zhang Y . HBx-induced S100A9 in NF-kappaB dependent manner promotes growth and metastasis of hepatocellular carcinoma cells. Cell Death Dis (2018) 9(6):629. doi: 10.1038/s41419-018-0512-2 29795379 PMC5967311

[28] Lou Y Yu Y Xu X Zhou S Shen H Fan T . Long non-coding RNA LUCAT1 promotes tumorigenesis by inhibiting ANXA2 phosphorylation in hepatocellular carcinoma. J Cell Mol Med (2019) 23(3):1873–84. doi: 10.1111/jcmm.14088 PMC6378214 30588744

[29] Sergeeva O Kepe V Zhang Y Miller-Atkins GA Keynon JD Iyer R . [(18)F] clofarabine for PET imaging of hepatocellular carcinoma. Cancers (2019) 11(11):1748. doi: 10.3390/cancers11111748 PMC6896045 31703407

[30] Dietrich P Gaza A Wormser L Fritz V Hellerbrand C Bosserhoff AK . Neuroblastoma RAS viral oncogene homolog (NRAS) is a novel prognostic marker and contributes to sorafenib resistance in hepatocellular carcinoma. Neoplasia (New York NY) (2019) 21(3):257–68. doi: 10.1016/j.neo.2018.11.011 PMC6370713 30685691

[31] Huntzicker EG Hotzel K Choy L Che L Ross J Pau G . Differential effects of targeting notch receptors in a mouse model of liver cancer. Hepatol (Baltimore Md) (2015) 61(3):942–52. doi: 10.1002/hep.27566 PMC4447303 25311838

[32] Wen Y Zhou X Lu M He M Tian Y Liu L . Bclaf1 promotes angiogenesis by regulating HIF-1alpha transcription in hepatocellular carcinoma. Oncogene (2019) 38(11):1845–59. doi: 10.1038/s41388-018-0552-1 PMC6462866 30367150

[33] Miao S Wang SM Cheng X Li YF Zhang QS Li G . Erythropoietin promoted the proliferation of hepatocellular carcinoma through hypoxia induced translocation of its specific receptor. Cancer Cell Int (2017) 17:119. doi: 10.1186/s12935-017-0494-7 29238266 PMC5725980

[34] Ng L Kwan V Chow A Yau TC Poon RT Pang R . Overexpression of Pin1 and rho signaling partners correlates with metastatic behavior and poor recurrence-free survival of hepatocellular carcinoma patients. BMC Cancer (2019) 19(1):713. doi: 10.1186/s12885-019-5919-3 31324164 PMC6642482

[35] Chen X Zhang S Wang Z Wang F Cao X Wu Q . Supervillin promotes epithelial-mesenchymal transition and metastasis of hepatocellular carcinoma in hypoxia *via* activation of the RhoA/ROCK-ERK/p38 pathway. J Exp Clin Cancer Research: CR (2018) 37(1):128. doi: 10.1186/s13046-018-0682-x 29954442 PMC6025706

[36] Li T Fan J Wang B Traugh N Chen Q Liu JS . TIMER: A web server for comprehensive analysis of tumor-infiltrating immune cells. Cancer Res (2017) 77(21):e108-e110. doi: 10.1158/1538-7445.CRC16-B11 29092952 PMC6042652

[37] Korbecki J Kojder K Simińska D Bohatyrewicz R Gutowska I Chlubek D . CC chemokines in a tumor: A review of pro-cancer and anti-cancer properties of the ligands of receptors CCR1, CCR2, CCR3, and CCR4. Int J Mol Sci (2020) 21(21):8412. doi: 10.3390/ijms21218412 PMC7665155 33182504

[38] Attia JVD Dessens CE van de Water R Houvast RD Kuppen PJK Krijgsman D . The molecular and functional characteristics of HLA-G and the interaction with its receptors: Where to intervene for cancer immunotherapy? Int J Mol Sci (2020) 21(22):8678. doi: 10.3390/ijms21228678 PMC7698525 33213057

[39] Strous GJ Almeida ADS Putters J Schantl J Sedek M Slotman JA . Growth hormone receptor regulation in cancer and chronic diseases. Front Endocrinol (2020) 11:597573. doi: 10.3389/fendo.2020.597573 PMC7708378 33312162

[40] Pan JH Zhou H Cooper L Huang JL Zhu SB Zhao XX . LAYN is a prognostic biomarker and correlated with immune infiltrates in gastric and colon cancers. Front Immunol (2019) 10:6. doi: 10.3389/fimmu.2019.00006 30761122 PMC6362421

